# Cross-species validation of a 6-miRNA blood signature for Parkinson’s disease: from MPTP mice to human PBMC and serum exosomes

**DOI:** 10.3389/fneur.2025.1704976

**Published:** 2025-11-26

**Authors:** Nagyum Jung, Seung-Nam Kim

**Affiliations:** 1 College of Korean Medicine, Dongguk University, Goyang, Republic of Korea; 2 Mangwondong Korean Medicine Clinic, Seoul, Republic of Korea

**Keywords:** Parkinson’s disease, microRNA biomarkers, blood-based biomarker, PBMC, serum exosomes, cross-species validation, early detection, precision medicine

## Abstract

**Introduction:**

Early detection of Parkinson’s disease (PD) remains challenging due to the lack of reliable blood-based biomarkers. While microRNAs (miRNAs) show promise as circulating biomarkers, translating preclinical discoveries to clinically applicable panels requires rigorous validation across platforms and populations.

**Methods:**

We performed temporal miRNA profiling in an acute MPTP mouse model (day 0 vs. day 5, *n* = 4 per group) using limma differential expression analysis with FDR correction. To address high-dimensional small-sample challenges, we employed global permutation testing and stability selection with elastic net regularization over 2,000 iterations. A compact miRNA panel was derived and validated in three independent human cohorts: GSE16658 (PBMC, *n* = 32), GSE269776 (serum exosomes 2021, *n* = 76), and GSE269775 (serum exosomes 2020, *n* = 100). Performance was assessed using ROC analysis with permutation-based *p*-values.

**Results:**

Seventeen miRNAs showed significant time-dependent changes in MPTP-treated mice (FDR <0.05), with 15 down-regulated and 2 up-regulated at day 5. Stability selection identified a 6-miRNA panel comprising miR-92b, miR-133a, miR-326, miR-125b, miR-148a, and miR-30b. External validation demonstrated consistent discriminative performance across platforms: GSE16658 AUC = 0.696 (*p* = 0.060), GSE269776 AUC = 0.791 (*p* < 0.001), and GSE269775 AUC = 0.725 (*p* < 0.001).

**Discussion:**

The signature showed platform-agnostic stability, performing comparably in PBMC and serum exosomes despite biological and technical differences. A 6-miRNA signature derived from acute MPTP response translates effectively to human blood samples, demonstrating reproducible PD discrimination across multiple platforms. The compact panel size and cross-platform compatibility support its potential for clinical biomarker development. By integrating AI-enhanced feature selection and permutation-based validation, this study provides a reproducible framework for biomarker discovery and a foundation for future early detection and precision medicine in Parkinson’s disease.

## 1 Introduction

Parkinson’s disease (PD) affects over 10 million people worldwide, yet its diagnosis remains heavily dependent on clinical observation of motor symptoms that typically manifest only after substantial dopaminergic neuronal loss has occurred (1, 2). This diagnostic delay represents a critical missed opportunity for early intervention, as disease-modifying therapies are most likely to be effective during prodromal stages when neurodegeneration is less advanced (3). Current diagnostic approaches lack objective molecular markers that can reliably detect PD before the onset of classical motor features, highlighting an urgent need for blood-based biomarkers that could enable earlier detection and monitoring of disease progression (4, 5).

MicroRNAs (miRNAs) have emerged as promising candidates for neurological biomarkers due to their stability in circulation, tissue-specific expression patterns, and roles in regulating key cellular processes implicated in neurodegeneration (6, 7). Several studies have identified altered miRNA profiles in PD patients’ blood, cerebrospinal fluid, and brain tissue, with individual miRNAs showing associations with disease severity and progression (8, 9). However, the field faces significant challenges in translating these discoveries into clinically applicable diagnostic tools. Most reported miRNA signatures lack validation across independent cohorts, suffer from platform-specific biases, or rely on large panels that are impractical for routine clinical use (10, 11).

The development of robust miRNA biomarkers requires a systematic approach that addresses both biological and methodological challenges. From a biological perspective, the heterogeneity of PD pathogenesis and the influence of confounding factors such as age, medication, and comorbidities complicate the identification of disease-specific signatures (12). Methodologically, the high-dimensional nature of miRNA data combined with typically small clinical samples creates substantial risks of overfitting and false discovery (13). These limitations have contributed to poor reproducibility across studies and the failure of many proposed miRNA biomarkers to advance beyond initial discovery phases (14).

Preclinical models offer valuable opportunities to identify and characterize miRNA responses under controlled conditions, potentially revealing signatures that translate to human disease. The 1-methyl-4-phenyl-1,2,3,6-tetrahydropyridine (MPTP) mouse model has been extensively used to study PD pathogenesis, as MPTP selectively targets dopaminergic neurons in the substantia nigra through mechanisms involving mitochondrial dysfunction and oxidative stress that mirror key aspects of human PD (15, 16). Acute MPTP administration induces rapid dopaminergic degeneration followed by partial recovery, providing a temporal window to capture both injury and compensatory responses (17). This time-course framework enables the identification of miRNA changes that are specifically associated with dopaminergic neuronal stress rather than general inflammatory or aging processes.

The translation of preclinical miRNA signatures to human biomarkers requires rigorous validation strategies that account for cross-species differences, platform variations, and population heterogeneity. Traditional approaches often fail because they rely on univariate statistical methods that cannot handle the complexity of miRNA data or because they lack sufficient external validation (18). Machine learning approaches, particularly those incorporating stability selection and robust cross-validation, offer more principled methods for deriving compact signatures that are less susceptible to overfitting (19, 20). However, the ultimate test of any biomarker is its performance across independent populations and measurement platforms, which remains the major bottleneck in biomarker development (21).

Recent advances in sample preparation and measurement technologies have enabled more standardized approaches to circulating miRNA analysis, including the use of serum exosomes that may provide more stable and tissue-relevant miRNA populations compared to total plasma or serum (22, 23). The availability of multiple large-scale datasets from different platforms and populations creates unprecedented opportunities for rigorous external validation of miRNA signatures, provided that appropriate statistical methods are employed to account for technical and biological heterogeneity (24).

In this study, we sought to develop a compact miRNA signature for PD discrimination using a systematic translational approach. We began with temporal miRNA profiling in an acute MPTP mouse model to identify signatures associated with dopaminergic neuronal stress. To address high-dimensional small-sample challenges, we employed global permutation testing to establish statistical significance and stability selection with elastic net regularization to derive a robust, compact miRNA panel. We then validated this signature across three independent human cohorts representing different sample types (peripheral blood mononuclear cells and serum exosomes) and populations, using permutation-based statistical testing to ensure robust performance assessment. Our goal was to determine whether a systematically derived miRNA signature could demonstrate reproducible discriminative performance across platforms and populations, thereby establishing a foundation for future clinical biomarker development.

## 2 Methods

### 2.1 Animals and sample collection

Male C57BL/6 mice (Orient-Bio Co., Republic of Korea), 8 weeks of age and weighing 20–25 g each, were used in this study. All animals were maintained on a 12/12 h light/dark cycle with ad libitum access to food and water. All experiments were approved by the Dongguk University Animal Care Committee for Animal Welfare (DGUIACUC-2018-022-2) and conducted according to institutional guidelines. Mice were randomly assigned to control or MPTP treatment groups (*n* = 4 per group per timepoint). MPTP (20 mg/kg; Sigma-Aldrich, United States) was administered intraperitoneally four times at 2-h intervals, while control animals received equivalent saline injections. This MPTP protocol has been extensively validated in our laboratory. In a separate cohort (*n* = 6 per group) using the identical protocol, we confirmed motor deficits at day 5 post-MPTP using the rotarod test, with MPTP-treated mice showing significantly reduced latency to fall compared to controls (data not shown). Behavioral testing was not performed in the miRNA profiling cohort to avoid potential stress-induced alterations in circulating miRNA levels. Blood samples were collected immediately after the final injection (day 0; D0) or 5 days post-injection (day 5; D5) to capture both acute and progressive responses to MPTP treatment. Serum was prepared by centrifugation at 1,200 × g for 15 min at 4 °C.

### 2.2 miRNA expression profiling

Total RNA was extracted from serum samples using the miRNeasy Serum/Plasma Kit (Qiagen, United States) following the manufacturer’s instructions. RNA purity and integrity were assessed using the ND-1000 Spectrophotometer (NanoDrop, Thermo Scientific, United States) and Agilent 2100 Bioanalyzer (Agilent Technologies, United States). miRNA expression profiling was performed using the Affymetrix GeneChip miRNA 4.0 array according to the manufacturer’s protocol. RNA samples (10 ng) were labeled using the FlashTag^™^ Biotin RNA Labeling Kit (Genisphere, United States), followed by quantification and fractionation. Labeled RNA was heated at 100 °C for 5 min, then 45 °C for 5 min before hybridization. Array hybridization was performed with agitation at 60 rpm for 16 h at 48 °C on the Affymetrix GeneChip platform. Chips were subsequently washed and stained using the Affymetrix Fluidics Station 450, then scanned using the Affymetrix GCS3000 scanner. Signal values were analyzed using Affymetrix GeneChip Command Console software, generating expression data for 3,163 miRNA probes across 16 samples (4 biological replicates × 2 groups × 2 timepoints). Raw expression data underwent log transformation and normalization. Sample quality was assessed using principal component analysis and summary statistics. Potential hemolysis contamination was evaluated using the miR-23a to miR-451 ratio as a proxy marker.

### 2.3 Statistical analysis of MPTP-responsive miRNAs

**Figure 1:**
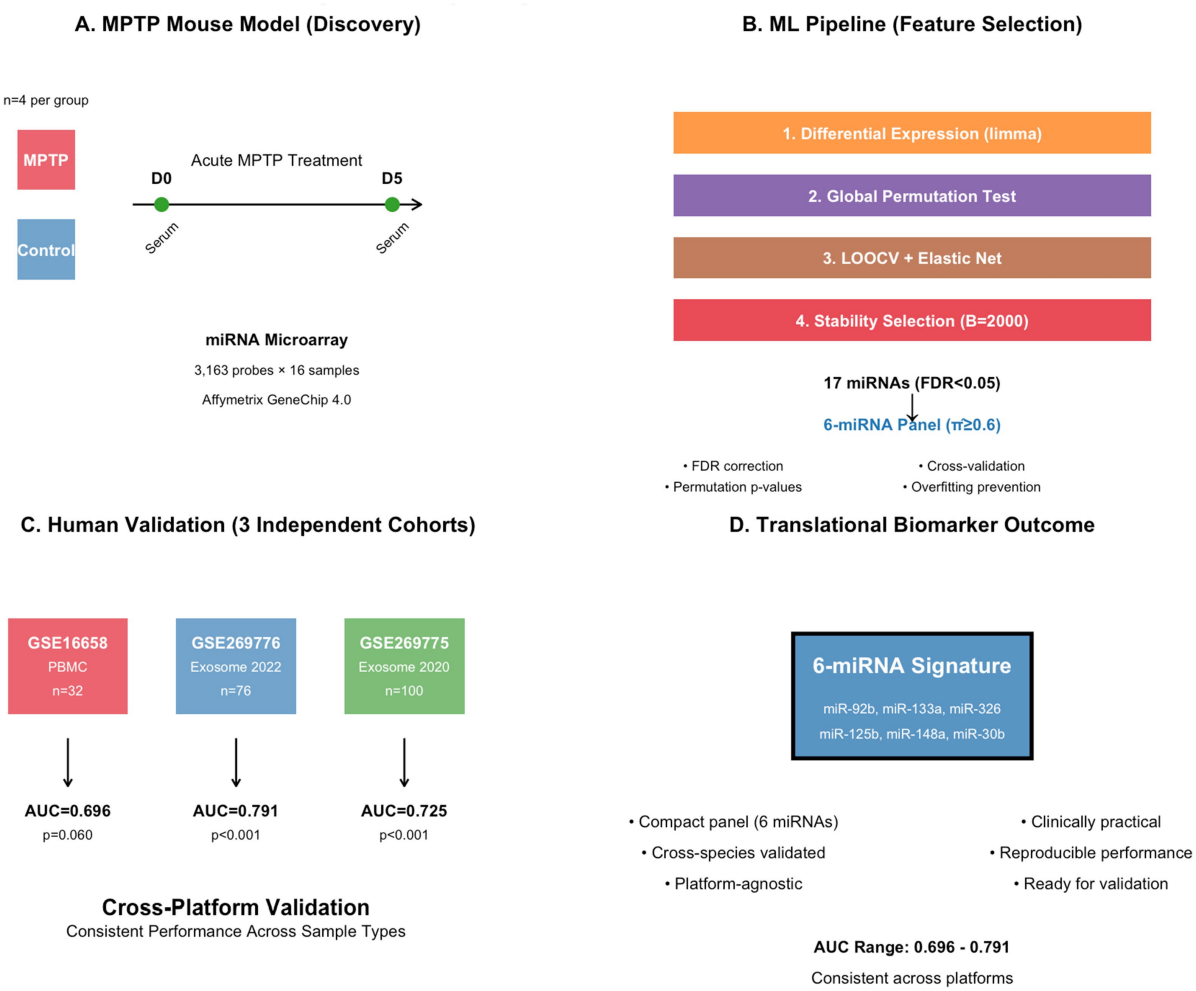
Study design and workflow for cross-species miRNA biomarker development comprehensive translational pipeline from preclinical discovery to clinical validation. **(A)** MPTP mouse model experimental design with acute treatment protocol and temporal sampling strategy (D0 baseline, D5 post-treatment). **(B)** Multi-step machine learning pipeline incorporating differential expression analysis (limma), statistical validation (global permutation testing), cross-validation (LOOCV + elastic net), and robust feature selection (stability selection over 2,000 iterations). **(C)** External validation across three independent human cohorts representing different sample types (PBMC vs. serum exosomes) and collection periods. **(D)** Final translational outcome showing cross-platform validated 6-miRNA signature with consistent discriminative performance.

The study design is illustrated in Figure 1. Differential expression analysis was performed using the limma package with a linear model incorporating group (Control vs. MPTP), time (D0 vs. D5), and group-by-time interaction terms. The primary contrast of interest was the time effect within the MPTP group (D5 vs. D0), which captures miRNA changes specifically associated with MPTP treatment (Figure 1A). Multiple testing correction was applied using the Benjamini–Hochberg false discovery rate (FDR) method, with significance defined as FDR <0.05. To address the high-dimensional small-sample problem inherent in miRNA data (3,163 features vs. 16 samples), we implemented global permutation testing. This approach involved permuting time labels within each treatment group 5,000 times and calculating a global test statistic based on the sum of squared *t*-statistics, providing a more robust assessment of overall significance compared to individual feature testing.

### 2.4 Machine learning pipeline for signature development

Feature selection and model development focused on the acute phase (day 0 samples only) to create a signature that could distinguish MPTP-treated from control mice. The analysis pipeline incorporated several strategies to prevent overfitting and ensure robustness (Figure 1B). First, we applied univariate filtering to select the top 20 miRNAs based on absolute t-statistic values within the training set. We then employed leave-one-out cross-validation (LOOCV) combined with elastic net regularization, testing alpha parameters of 0.1, 0.5, and 0.9 with lambda values selected via internal cross-validation. To account for class imbalance in the small sample, we used stratified fold assignment to ensure balanced representation in cross-validation.

Stability selection was performed over 2,000 iterations, with each iteration involving random subsampling of 2 samples per class (4 total samples) for training. Within each iteration, we applied the same preprocessing, filtering, and elastic net modeling pipeline. The selection probability threshold of $\overset{⌢}{\pi }$ ≥ 0.6 was adopted following established stability selection frameworks (20), which recommend thresholds between 0.6 and 0.9 as an optimal compromise between feature selection stability and control of false discoveries. This criterion has been widely applied in high-dimensional omics analyses to ensure reproducibility and minimize overfitting.

Performance evaluation included permutation testing with 5,000 label permutations to establish empirical *p*-values for the observed AUC values. This permutation-based approach is particularly important for small sample sizes where asymptotic statistical assumptions may not hold.

### 2.5 Human cohort validation

Three independent human datasets were used for external validation: GSE16658 (peripheral blood mononuclear cells, *n* = 32), GSE269776 (serum exosomes 2022, *n* = 76), and GSE269775 (serum exosomes 2020, *n* = 100). These cohorts represent different sample types, collection years, and populations, providing robust tests of signature generalizability (Figure 1C).

For GSE16658, we obtained expression data and phenotype information from the Gene Expression Omnibus. Samples were classified as control or PD based on the “disease state” annotation. Platform-specific miRNA identifiers were mapped to human miRNA names using the GPL7722 annotation, retaining only probes corresponding to human miRNAs (hsa-miR prefix) and excluding viral sequences.

For the serum exosome datasets (GSE269776 and GSE269775), raw count matrices were obtained from Supplementary Excel files. Sample labels were parsed from column headers for GSE269776 (PD vs. Control prefixes) and inferred from sample numbering for GSE269775 (samples 001–050 as PD, 051–100 as Control). miRNA identifiers were standardized by extracting mature miRNA sequences from complex probe names and converting to lowercase format.

### 2.6 Cross-platform miRNA name harmonization

To ensure consistent miRNA identification across mouse and human platforms, we implemented a systematic name normalization procedure. Mouse miRNA names (mmu-miR prefix) were converted to human equivalents (hsa-miR prefix), with subsequent conversion to lowercase. Arm-specific information (3p/5p suffixes) was removed to create “core” miRNA names that could be matched across different annotation systems. When multiple probes mapped to the same core miRNA name, expression values were averaged.

Sequence analysis revealed complete identity between mouse and human orthologues for 5 of 6 signature miRNAs (miR-92b, miR-133a, miR-125b, miR-148a, and miR-30b). For miR-326, the mouse signature contained the 5p arm (mmu-miR-326-5p) while human datasets predominantly featured the 3p arm (hsa-miR-326), representing different mature miRNAs derived from the same precursor. Both arms were retained in the analysis as they derive from the same genomic locus and regulatory context. This approach maximizes the overlap between signatures derived from mouse data and those measurable in human datasets while maintaining biological relevance (Table 1).

**Table 1:** Cross-species sequence comparison of the 6-miRNA signature between mouse and human orthologues.

mouse_miRNA	human_miRNA	mouse_seq	human_seq	seed_mouse	seed_human	seed_match	Notes
mmu-miR-92b-3p	hsa-miR-92b-3p	UAUUGCACUCGUCCCGGCCUCC	UAUUGCACUCGUCCCGGCCUCC	AUUGCAC	AUUGCAC	Yes	Identical
mmu-miR-133a-3p	hsa-miR-133a-3p	UUUGGUCCCCUUCAACCAGCUG	UUUGGUCCCCUUCAACCAGCUG	UUGGUCC	UUGGUCC	Yes	Identical
mmu-miR-326-5p	hsa-miR-326	GGGGGCAGGGCCUUUGUGAAGGCG	CCUCUGGGCCCUUCCUCCAG	GGGGCAG	CUCUGGG	No	Not identical
mmu-miR-125b-5p	hsa-miR-125b-5p	UCCCUGAGACCCUAACUUGUGA	UCCCUGAGACCCUAACUUGUGA	CCCUGAG	CCCUGAG	Yes	Identical
mmu-miR-148a-3p	hsa-miR-148a-3p	UCAGUGCACUACAGAACUUUGU	UCAGUGCACUACAGAACUUUGU	CAGUGCA	CAGUGCA	Yes	Identical
mmu-miR-30b-5p	hsa-miR-30b-5p	UGUAAACAUCCUACACUCAGCU	UGUAAACAUCCUACACUCAGCU	GUAAACA	GUAAACA	Yes	Identical

Mouse and human miRNA sequences were compared to assess cross-species conservation. Seed sequences (nucleotides 2–8 from the 5′ end) were analyzed for functional similarity. Five of six miRNAs showed complete sequence identity between species, while miR-326 represents different mature arms (5p in mouse, 3p in human) from the same precursor, resulting in distinct seed sequences but shared genomic origin.

### 2.7 Signature scoring and validation

The 6-miRNA signature was applied to human cohorts using a standardized scoring approach (Figure 1D). For each cohort, expression values were *z*-score normalized across samples for each miRNA. The signature score was calculated as the mean of *z*-scores for down-regulated miRNAs (with sign reversal) minus the mean for up-regulated miRNAs.

Performance was evaluated using receiver operating characteristic (ROC) analysis to calculate area under the curve (AUC) values with 95% confidence intervals. Statistical significance was assessed using permutation testing with 5,000 label permutations, calculating empirical *p*-values as the proportion of permuted AUCs greater than or equal to the observed AUC. This non-parametric approach provides robust significance testing that does not rely on distributional assumptions and is particularly appropriate for the modest sample sizes in our validation cohorts.

### 2.8 Reproducibility and code availability

All analyses were performed using R version 4.0 or later with specific package versions recorded for reproducibility. Random number generator seeds were set consistently across all stochastic procedures (seed = 20,250,912). Analysis code and processed data are publicly available to ensure full reproducibility of results. This data can be found here: https://osf.io/vs4w9/ (doi: 10.17605/OSF.IO/VS4W9).

## 3 Results

### 3.1 Sample characteristics and MPTP-responsive miRNA identification

**Figure 2:**
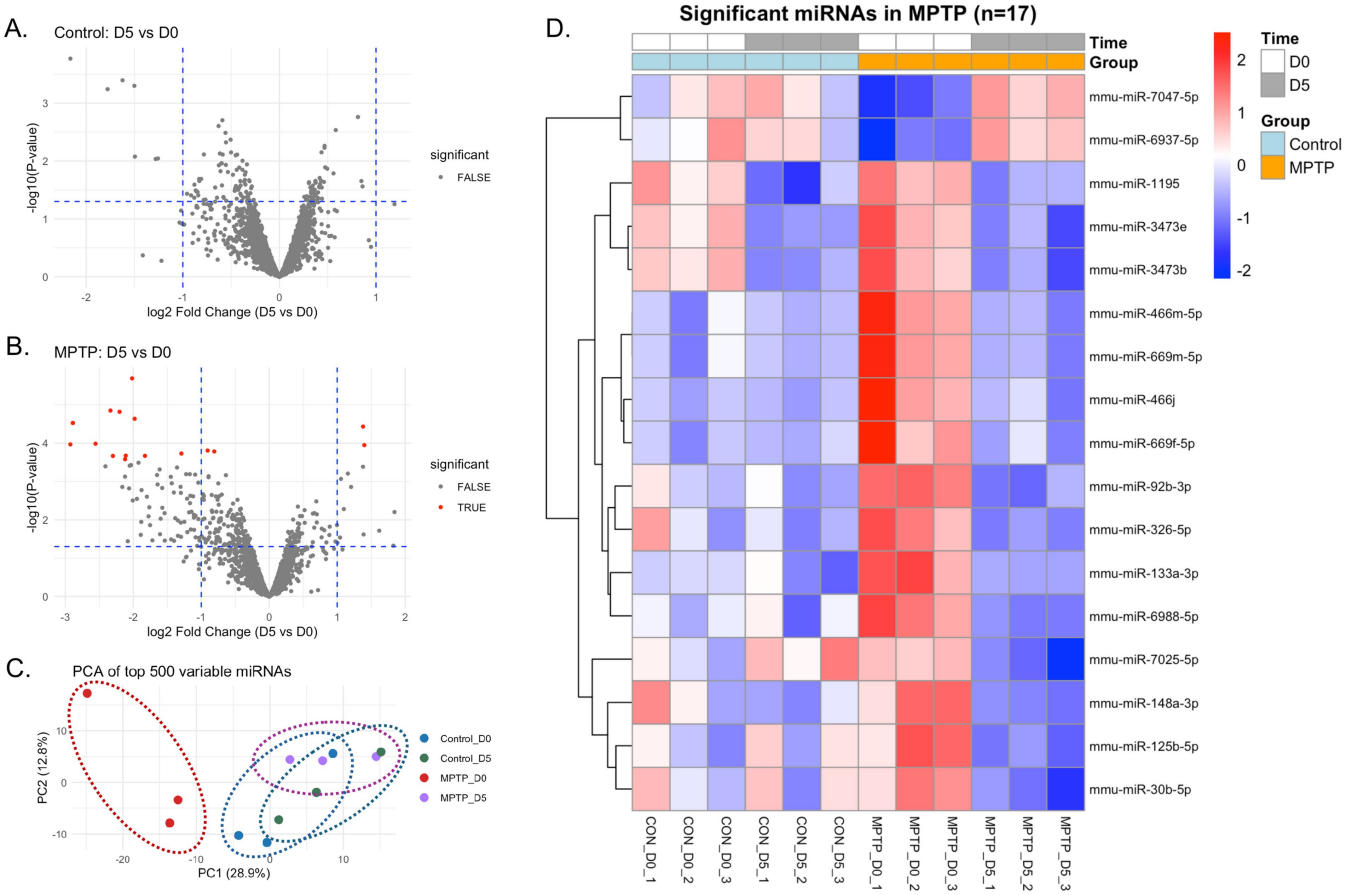
Differential expression analysis and discovery of MPTP-responsive miRNAs. Temporal miRNA expression changes following acute MPTP treatment. **(A,B)** Volcano plots comparing D5 vs. D0 expression in Control and MPTP groups, respectively. MPTP treatment resulted in 17 significantly altered miRNAs (FDR < 0.05), while Control showed minimal changes. **(C)** Principal component analysis (PCA) of the top 500 variable miRNAs. The first two principal components explained 28.9 and 12.8% of the total variance, respectively. Dashed ellipses were drawn to visually group samples by condition (Control_D0/D5 and MPTP_D0/D5), but do not represent statistical confidence intervals due to the limited sample size. **(D)** Heatmap of 17 significant miRNAs across all samples, demonstrating predominant down-regulation in MPTP-treated animals at D5.

The study included 16 serum samples from male C57BL/6 mice across two treatment groups (Control vs. MPTP) and two timepoints (D0 and D5), with 4 biological replicates per condition (Figures 2A,B). Principal component analysis of the top 500 most variable miRNAs revealed clear separation between treatment groups and timepoints, with PC1 explaining 28.9% of total variance (Figure 2C). Sample quality metrics indicated consistent RNA extraction efficiency across groups, with miR-23a to miR-451 ratios suggesting minimal hemolysis contamination (data not shown). All 16 samples passed quality control criteria and were retained for subsequent analysis.

Differential expression analysis using limma identified 17 miRNAs showing significant time-dependent changes in MPTP-treated mice (FDR <0.05), while no miRNAs met significance criteria for time effects in control animals. Of the 17 MPTP-responsive miRNAs, 15 were down-regulated and 2 were up-regulated at day 5 compared to day 0. The most significantly down-regulated miRNA was mmu-miR-92b-3p (logFC = −2.017, adj. *p* = 0.0063), followed by mmu-miR-3473e (logFC = −2.335, adj. *p* = 0.015) and mmu-miR-3473b (logFC = −2.202, adj. *p* = 0.015). Up-regulated miRNAs included mmu-miR-7047-5p (logFC = 1.381, adj. *p* = 0.016) and mmu-miR-6937-5p (logFC = 1.399, adj. *p* = 0.035). The predominance of down-regulated miRNAs suggests that MPTP treatment primarily leads to decreased miRNA expression at day 5, potentially reflecting cellular stress responses or compensatory mechanisms following acute dopaminergic insult (Figure 2D).

### 3.2 Stability selection and panel development

**Figure 3:**
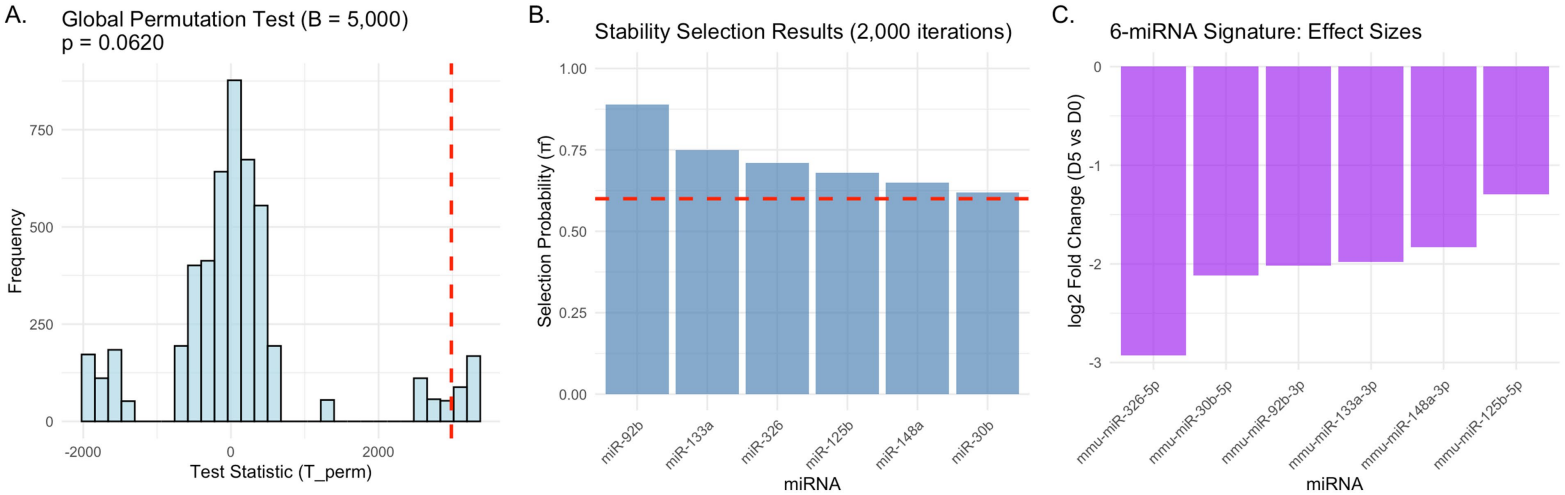
Machine learning pipeline and statistical validation. Statistical validation and feature selection for robust miRNA signature development. **(A)** Global permutation test comparing time effects between MPTP and Control groups (*B* = 5,000 permutations). Red dashed line indicates observed test statistic, with *p* = 0.062 showing marginal significance. **(B)** Stability selection results from 2,000 iterations identifying miRNAs with selection probability $\overset{⌢}{\pi }$ ≥ 0.6 (red dashed threshold). Six miRNAs met the stability criterion for inclusion in the final signature. **(C)** Effect sizes of the 6-miRNA signature showing log2 fold changes (D5 vs. D0) in MPTP-treated mice, with predominant down-regulation consistent with the signature scoring approach.

Global permutation testing provided additional validation of these findings. The test statistic comparing time effects between MPTP and control groups yielded a one-sided *p*-value of 0.062 (*B* = 5,000 permutations), showing a trend toward significance that suggests the observed pattern of miRNA changes in MPTP-treated animals may reflect genuine biological effects rather than random variation. This result addresses concerns about multiple testing in high-dimensional data and provides supportive evidence for the biological relevance of the identified miRNA signature (Figure 3A).

Machine learning analysis focused on day 0 samples to develop a signature capable of distinguishing MPTP-treated from control mice. Leave-one-out cross-validation with elastic net regularization achieved a mean AUC of 0.92 across folds, indicating strong discriminative performance. However, the small sample size (*n* = 8) necessitated additional validation through stability selection to ensure robust feature selection.

Stability selection over 2,000 iterations with random subsampling identified miRNAs with consistent selection across multiple training sets. Six miRNAs achieved selection probabilities $\overset{⌢}{\pi }$ ≥ 0.6: mmu-miR-92b-3p ( $\overset{⌢}{\pi }$ = 0.89), mmu-miR-133a-3p ( $\overset{⌢}{\pi }$ = 0.75), mmu-miR-326-5p ( $\overset{⌢}{\pi }$ = 0.71), mmu-miR-125b-5p ( $\overset{⌢}{\pi }$ = 0.68), mmu-miR-148a-3p ( $\overset{⌢}{\pi }$ = 0.65), and mmu-miR-30b-5p ( $\overset{⌢}{\pi }$ = 0.62) (Figure 3B). All six miRNAs were among the down-regulated features in the temporal analysis, creating a coherent signature where decreased expression levels indicate MPTP-associated changes (Figure 3C).

Permutation testing of the stability selection procedure yielded an empirical *p*-value of 0.032 (*B* = 2,000), indicating that the observed selection frequencies were significantly higher than expected under the null hypothesis of random feature selection. This result provides confidence that the 6-miRNA panel represents genuine biological signal rather than statistical artifact.

### 3.3 External validation in human cohorts

The 6-miRNA signature was evaluated in three independent human datasets representing different sample types and populations. Cross-platform miRNA name harmonization successfully mapped all six mouse miRNAs to human orthologues, with complete sequence identity observed for five miRNAs (miR-92b, miR-133a, miR-125b, miR-148a, and miR-30b). For miR-326, the mouse signature contained the 5p arm while human datasets featured the 3p arm, representing different mature miRNAs from the same precursor. Platform-specific probe mapping resulted in successful detection of all six miRNAs across the three human datasets.

**Figure 4:**
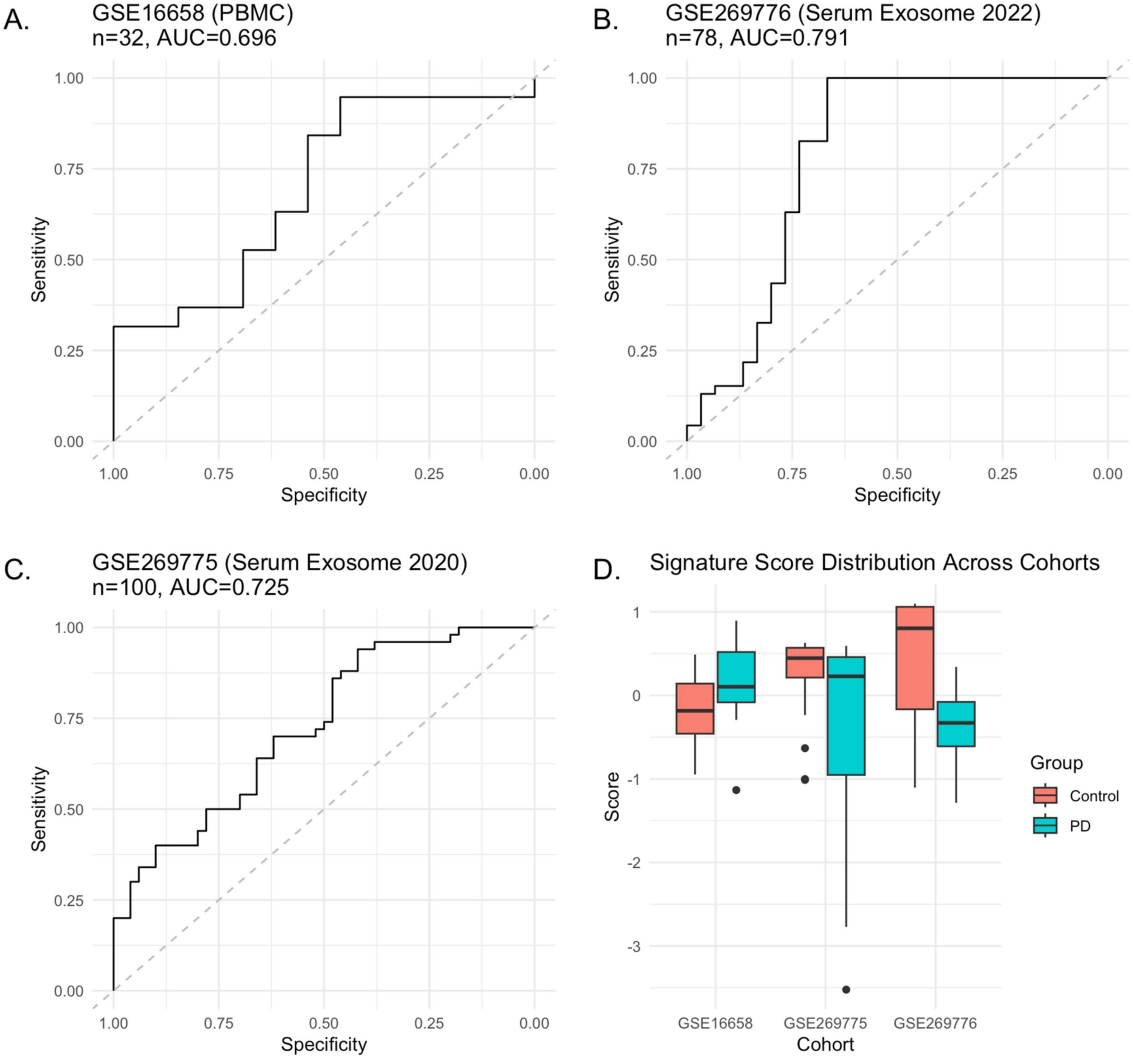
External validation of the 6-miRNA signature in human cohorts. Cross-platform validation demonstrating reproducible discriminative performance across independent human datasets. **(A–C)** ROC curves for three validation cohorts: GSE16658 (PBMC), GSE269776 (serum exosome 2022), and GSE269775 (serum exosome 2020). Dashed diagonal lines represent random classifier performance. AUC values demonstrate consistent discriminative ability across different sample types and platforms. **(D)** Signature score distributions showing consistent directional patterns across all cohorts, with Control samples generally exhibiting higher scores than PD samples, matching the down-regulation pattern observed in the MPTP mouse model.

The diagnostic performance of the 6-miRNA signature was systematically evaluated across all cohorts, with detailed metrics including AUC, 95% confidence intervals, sensitivity, specificity, and optimal thresholds summarized in Supplementary Table S1. In GSE16658 (peripheral blood mononuclear cells, *n* = 32), the signature achieved an AUC of 0.696 (95% CI: 0.51–0.88) for discriminating PD patients (*n* = 19) from controls (*n* = 13). At the optimal threshold of −0.30, the signature demonstrated a sensitivity of 95% and specificity of 46%. Permutation testing yielded a *p*-value of 0.060, indicating marginal statistical significance. Signature scores showed the expected pattern, with controls exhibiting lower mean scores (−0.19 ± 0.44) compared to PD patients (0.13 ± 0.47), consistent with the down-regulation pattern observed in MPTP-treated mice (Figure 4A).

Validation in serum exosome datasets demonstrated stronger performance. GSE269776 (2022 cohort, *n* = 76) achieved an AUC of 0.791 (95% CI: 0.68–0.90) with controls (*n* = 30) showing higher signature scores (0.45 ± 0.74) than PD patients (*n* = 46, −0.34 ± 0.40). At the optimal threshold of −0.52, the signature achieved 100% sensitivity and 69% specificity. Permutation testing confirmed statistical significance (*p* < 0.001) (Figure 4B). Similarly, GSE269775 (2020 cohort, *n* = 100) yielded an AUC of 0.725 (95% CI: 0.62–0.83) with controls (*n* = 50) scoring higher (0.31 ± 0.39) than PD patients (*n* = 50, −0.31 ± 1.04), achieving 94% sensitivity and 42% specificity at the optimal threshold of −0.37, also achieving statistical significance (*p* < 0.001) (Figure 4C).

Statistical significance was achieved in 2 of 3 cohorts using conservative permutation-based testing (GSE269776 and GSE269775, both *p* < 0.001), while GSE16658 approached significance (*p* = 0.060). The signature demonstrated moderate-to-high discriminative performance across different sample types, measurement platforms, and populations, with AUC values ranging from 0.696 to 0.791 (Figure 4D). These performance metrics, particularly the high sensitivity (94–100%) observed in the serum exosome cohorts, are consistent with acceptable discrimination thresholds for biomarker studies in complex multifactorial neurodegenerative diseases, where biological and technical heterogeneity typically limit classification power (25).

## 4 Discussion

This study demonstrates that a compact 6-miRNA signature derived from acute MPTP response can provide reproducible discrimination of PD samples across multiple human cohorts and measurement platforms. The systematic translational approach, combining rigorous statistical methods with external validation, addresses key limitations that have hindered previous miRNA biomarker studies.

The predominant down-regulation of miRNAs at day 5 post-MPTP treatment suggests a coordinated response to dopaminergic stress that involves decreased miRNA-mediated gene regulation. This pattern may reflect cellular adaptation mechanisms, as reduced miRNA expression could allow increased translation of protective proteins during recovery from acute injury (26). The specific miRNAs identified have established roles in neuronal function and stress responses. miR-125b regulates neuroinflammation and has been implicated in neurodegenerative diseases (27), while miR-133b controls dopaminergic neuron maturation and survival (28). miR-30b and miR-148a are involved in autophagy regulation and protein aggregation, processes central to PD pathogenesis (29, 30).

The superior performance of serum exosome-based measurements compared to peripheral blood mononuclear cells likely reflects the enriched and stable miRNA content of exosomal preparations. Exosomes protect miRNAs from degradation and may provide more tissue-relevant signatures compared to total plasma miRNAs (31). This finding supports the growing emphasis on exosome-based biomarker development and suggests that standardized exosome isolation protocols could improve the clinical utility of miRNA signatures.

Cross-platform robustness represents a critical advancement for miRNA biomarker translation. The consistent performance across datasets spanning different years, populations, and technologies demonstrates that the signature captures conserved biological processes rather than platform-specific artifacts. The successful cross-species translation from mouse to human suggests that MPTP-induced miRNA changes reflect fundamental responses to dopaminergic stress that are preserved across species. Notably, the signature maintained discriminative performance despite one miRNA (miR-326) representing different mature arms across species, suggesting that pathway-level conservation rather than precise sequence identity drives the discriminative signal.

The observed sensitivity-specificity trade-off across validation cohorts reflects different optimal threshold selections and may provide insights for future clinical implementation strategies. The serum exosome-based measurements (GSE269776 and GSE269775) demonstrated particularly high sensitivity (94–100%), which is clinically advantageous for screening applications where false negatives are more costly than false positives. The moderate specificity (42–69%) suggests that the signature may benefit from combination with complementary biomarkers or clinical features to enhance positive predictive value in future clinical implementations. This pattern is consistent with the early-stage biomarker development paradigm, where prioritizing sensitivity enables broader capture of at-risk individuals for subsequent confirmatory testing.

Several limitations must be acknowledged. First, although the 6-miRNA signature was derived from robust microarray profiling and validated across multiple human datasets, additional validation using independent quantification techniques such as quantitative PCR or digital droplet PCR is warranted to confirm cross-platform reproducibility at the technical level. Second, repeated handling and intraperitoneal injections in the mouse model may have induced stress-related or systemic inflammatory responses that could influence circulating miRNA levels independently of dopaminergic injury, potentially confounding the identification of PD-specific signatures. Third, only male mice were used in the MPTP model to minimize estrogen-related variability in MPTP susceptibility (32), which may restrict generalizability and overlook sex-specific miRNA responses that could be relevant to human Parkinson’s disease, where sex differences in disease presentation are well-documented (33). The mouse model employed acute MPTP treatment rather than chronic exposure, which may not fully recapitulate the progressive nature of human PD (15). The human validation cohorts represent cross-sectional rather than longitudinal designs, limiting conclusions about the signature’s utility for early detection or disease monitoring. Sample sizes in individual cohorts were modest, and the marginal significance in GSE16658 highlights the importance of larger validation studies (14). Additionally, clinical covariates such as medication status, disease duration, and severity scores were not consistently available across datasets, preventing assessment of these potentially confounding factors.

The statistical approach addresses but does not eliminate concerns about high-dimensional data analysis with small samples. While stability selection and permutation testing provide principled methods for feature selection and significance assessment (19, 20), the fundamental challenge of limited biological replicates remains. Future studies should prioritize larger sample sizes, incorporate both sexes, include chronic or progressive PD models, perform independent technical validation using orthogonal methods, and ensure standardized clinical characterization to enable more robust and generalizable biomarker development.

The translational pathway from preclinical models to clinical biomarkers requires systematic validation approaches that extend beyond single-cohort discovery studies. Our results suggest that mouse MPTP models can provide valuable starting points for human biomarker identification, but emphasize the need for rigorous external validation across multiple platforms and populations (17). The compact nature of the 6-miRNA panel supports practical clinical implementation, as smaller signatures are more amenable to standardized measurement protocols and regulatory approval processes.

Future research should focus on prospective validation in well-characterized clinical cohorts with longitudinal follow-up to assess the signature’s performance in prodromal stages and its utility for disease monitoring. Integration with other biomarker modalities, including protein markers and imaging measures, may provide complementary information that enhances diagnostic accuracy (21). Mechanistic studies exploring the functional roles of the signature miRNAs in dopaminergic neuron biology could inform therapeutic target identification and deepen understanding of PD pathogenesis.

In conclusion, this study establishes a systematic framework for translating preclinical miRNA discoveries to human biomarker applications. By incorporating AI-driven stability selection, elastic net regularization, and permutation-based validation, we demonstrate how machine learning can overcome high-dimensional small-sample challenges and enhance reproducibility. The resulting 6-miRNA signature shows reproducible discriminative performance across platforms and populations, supporting its potential as a clinically feasible blood-based diagnostic tool for PD. While current results represent discrimination rather than early detection, the integration of AI-enhanced biomarker discovery with cross-platform validation provides a strong foundation for future applications in early diagnosis and precision medicine.

## Data Availability

The original contributions presented in the study are publicly available. This data can be found here: https://osf.io/vs4w9/ (DOI: 10.17605/OSF.IO/VS4W9). Human cohort datasets are available in Gene Expression Omnibus: GSE16658, GSE269776, GSE269775.
