# Supplementary material for: Cross-species validation of a 6-miRNA blood signature for Parkinson’s disease: from MPTP mice to human PBMC and serum exosomes
Source: Front Neurol. 2025 Nov 26;16:1704976. doi: 10.3389/fneur.2025.1704976 (PMC12689404; doi:10.3389/fneur.2025.1704976)
Supplement: Supplementary file 1 [file Table_1.docx]

**Table S1. Summary of performance metrics for 6-miRNA signature across validation cohorts.**

| Cohort | Platform / Sample type | N (PD / Control) | AUC (95% CI) | p-value (perm.) | Sensitivity | Specificity | Threshold |
| --- | --- | --- | --- | --- | --- | --- | --- |
| GSE16658 | PBMC (Affymetrix GPL7722) | 32 (19 / 13) | 0.696 (0.51–0.88) | 0.060 | 0.95 | 0.46 | -0.3009443 |
| GSE269776 | Serum exosome (2022) | 76 (46 / 30) | 0.791 (0.68–0.90) | <0.001 | 1 | 0.69 | -0.5193434 |
| GSE269775 | Serum exosome (2020) | 100 (50 / 50) | 0.725 (0.62–0.83) | <0.001 | 0.94 | 0.42 | -0.3730627 |

*Optimal thresholds were determined by Youden’s J; 95% CIs were calculated using 2,000-bootstrap replicates; Permutation p-values were estimated with 5,000 label permutations.
